# Diagnostic Yield and Impact on Antimicrobial Management of 16S rRNA Testing of Clinical Specimens

**DOI:** 10.1128/spectrum.02094-22

**Published:** 2022-11-14

**Authors:** Pruke Eamsakulrat, Pitak Santanirand, Angsana Phuphuakrat

**Affiliations:** a Department of Medicine, Faculty of Medicine Ramathibodi Hospital, Mahidol University, Bangkok, Thailand; b Department of Pathology, Faculty of Medicine Ramathibodi Hospital, Mahidol University, Bangkok, Thailand; Quest Diagnostics

**Keywords:** 16S rRNA, antimicrobial management, clinical specimens, diagnostic yield, impact

## Abstract

16S rRNA gene sequencing is increasingly used in clinical practice for bacterial identification of clinical specimens. However, studies on its applicability to direct clinical specimens are limited. Here, we studied the diagnostic yield and impact of 16S rRNA gene sequencing from direct clinical specimens on antimicrobial management. Adult inpatients whose attending physician requested 16S rRNA gene sequencing and corresponding bacterial culture from a direct clinical specimen between January and December 2021 in a university hospital were prospectively included in this study. A total of 434 specimens from 374 patients were requested. Of these, 253 (58.3%) specimens were collected from patients whose final diagnosis indicated a bacterial infection, whereas 181 (41.7%) specimens were from nonbacterial infections. Using the final diagnosis as a “gold standard,” the sensitivity and specificity of 16S rRNA gene sequencing were 38.3% and 93.9%, respectively. Among the bacterial infection cases, the proportion of 16S rRNA gene sequencing-positive and culture-positive cases was 32.4%, and the proportion of sequencing-positive and culture-negative cases was 5.9%. The impact on antimicrobial management was evident in 10 (2.3%) specimens, which all resulted in the continuation of antibiotics. The impact on antimicrobial management was highest in skin and soft tissue infections, followed by bone and joint infections. In this study, the long turnaround time of 16S rRNA gene sequencing of clinical specimens was a limiting factor. In conclusion, the overall diagnostic yield of 16S rRNA gene sequencing in bacterial infection cases was fair, being useful in selected cases. Restrictions on test requests may improve test utilization in this setting.

**IMPORTANCE** 16S rRNA gene sequencing has been increasingly used in clinical practice. Using the final diagnosis as a gold standard, the sensitivity of 16S rRNA gene sequencing was fair. In the setting with no 16S rRNA gene sequencing test ordering restrictions, only small percentages of the test results had an impact on antimicrobial management. Restrictions on test requests should be developed to maximize the benefit of the test.

## INTRODUCTION

Infectious diseases caused by bacterial infections are common in clinical practice. Conventional bacterial culture and phenotypic testing help clinicians detect bacteria and adjust antimicrobial treatment accordingly. However, conventional biochemical tests cannot identify all pathogens. Various methods, such as matrix-assisted laser desorption ionization–time of flight mass spectrometry (MALDI-TOF MS), broad-range 16S rRNA gene sequencing, multiplex molecular panel sequencing, and next-generation sequencing, have been used to improve pathogen identification (1–7).

16S rRNA gene sequencing is more accurate than biochemical testing for the identification of specific bacterial species (8, 9). The utility of 16S rRNA gene sequencing for pathogen identification in routine diagnostic microbiology has been long established. This method can also identify difficult to culture or unculturable bacteria from direct clinical specimens (10–13). Various studies have aimed to apply 16S rRNA gene sequencing in clinical practice, such as in native vertebral osteomyelitis and infective endocarditis, but few studies have determined the value of this method in antimicrobial management (14–16).

Despite limited evidence, 16S rRNA gene sequencing has been introduced into clinical practice and its use has increased over time. Our study aimed to enhance knowledge on the impact of 16S rRNA gene sequencing for bacterial identification from direct clinical specimens on antimicrobial management.

## RESULTS

### Patient characteristics.

A total of 434 specimens from 374 patients were requested during the study period. The median (interquartile range [IQR]) age of the patients was 62 (52 to 74) years, and 208 patients (47.9%) were male. Nearly half (45.2%) of the patients were immunocompromised, including patients with hematologic malignancy (*n* = 57), solid malignancy (*n* = 56), autoimmune disease (*n* = 47), solid organ transplant (*n* = 10), AIDS (*n* = 7), adult-onset immunodeficiency (*n* = 7), and other causes (*n* = 12). The most common provisional diagnosis was skin and soft tissue infection (14.7%), followed by community-acquired pneumonia (CAP) (11.5%), hospital-acquired pneumonia/ventilator-associated pneumonia (HAP/VAP) (11.5%), septic arthritis (10.8%), meningitis (7.1%), and native vertebral osteomyelitis (6.5%).

There was a statistically significant difference in the proportions of provisional diagnoses between the 16S rRNA gene sequencing-positive group and the 16S rRNA gene sequencing-negative group (*P* < 0.001) (Table 1). The most common provisional diagnosis in the 16S rRNA gene sequencing-positive group was HAP/VAP (20.4%), followed by skin and soft tissue infection (18.5%) and CAP (17.6%), whereas in the 16S rRNA gene sequencing-negative group, the most common provisional diagnoses were skin and soft tissue infection (13.5%), septic arthritis (11.3%), and CAP (9.5%).

**TABLE 1:** Clinical characteristics and demographics of the patients

Parameter^*a*^	Result for:			*P* value
	All (*n* = 434)	16S rRNA sequencing		
		Positive (*n* = 108)	Negative (*n* = 326)	
Median (IQR) age, yr	62 (52–74)	66 (52–77)	61 (51–73)	0.059
Male, *n* (%)	208 (47.9)	63 (58.3)	145 (44.5)	0.012
Immune status, *n* (%)				0.863
Immunocompetent	238 (54.8)	60 (55.6)	178 (54.6)	
Immunocompromised	196 (45.2)	48 (44.4)	148 (45.4)	
Provisional diagnosis, *n* (%)				<0.001
Skin and soft tissue infection	64 (14.7)	20 (18.5)	44 (13.5)	
CAP	50 (11.5)	19 (17.6)	31 (9.5)	
HAP/VAP	50 (11.5)	22 (20.4)	28 (8.6)	
Septic arthritis	47 (10.8)	10 (9.3)	37 (11.3)	
Meningitis	31 (7.1)	0 (0.0)	31 (9.5)	
Native vertebral osteomyelitis	28 (6.5)	3 (2.8)	25 (7.7)	
Eye infection	24 (5.5)	5 (4.6)	19 (5.8)	
Prosthetic joint infection	22 (5.1)	3 (2.8)	19 (5.8)	
Parapneumonic effusion	21 (4.8)	1 (0.9)	20 (6.1)	
Osteomyelitis	18 (4.1)	6 (5.6)	12 (3.7)	
Postoperative meningitis	14 (3.2)	1 (0.9)	13 (4.0)	
Intra-abdominal collection	13 (3.0)	4 (3.7)	9 (2.8)	
Brain/epidural abscess	9 (2.1)	2 (1.9)	7 (2.1)	
Peri/myocarditis	9 (2.1)	0 (0.0)	9 (2.8)	
Liver abscess	6 (1.4)	5 (4.6)	1 (0.3)	
Bone marrow infection	5 (1.2)	0 (0.0)	5 (1.5)	
Lymphadenopathy	4 (0.9)	1 (0.9)	3 (0.9)	
Renal abscess	3 (0.7)	2 (1.9)	1 (0.3)	
Lung abscess	3 (0.7)	1 (0.9)	2 (0.6)	
Perinephric collection	3 (0.7)	2 (1.9)	1 (0.3)	
SBP	3 (0.7)	0 (0.0)	3 (0.9)	
Infective endocarditis	2 (0.5)	0 (0.0)	2 (0.6)	
Chorioamnionitis	2 (0.5)	0 (0.0)	2 (0.6)	
Aortitis	1 (0.2)	0 (0.0)	1 (0.3)	
Colitis	1 (0.2)	1 (0.9)	0 (0.0)	
Splenic abscess	1 (0.2)	0 (0.0)	1 (0.3)	
Previous exposure to antibiotic, *n* (%)				
Within 14 days	29 (6.7)	6 (5.6)	23 (7.1)	0.589
Within 90 days	59 (13.6)	17 (15.7)	42 (12.9)	0.453
Median (IQR) days of previous exposure to antibiotic within 90 days	20 (5–30)	21 (5–30)	13 (5–31)	0.511
Currently on antibiotic, *n* (%)^*b*^	294 (67.7)	81 (75.0)	213 (65.3)	0.063
Median (IQR) days currently on antibiotic	8 (3–18)	10 (5–21)	7 (2–16)	0.027
Specimen, *n* (%)				
Fluid	284 (65.4)	77 (71.3)	207 (63.5)	0.140
Tissue	150 (34.6)	31 (28.7)	119 (36.5)	
Gram stain positive for bacteria, *n* (%) (*n* = 410)	60 (14.6)	47/106 (44.3)	13/304 (4.3)	<0.001
Culture positive, *n* (%)	131 (30.2)	92 (85.2)	39 (12.0)	<0.001
Median (IQR) turnaround time, days	3 (2–10)	11 (9–13)	2 (1–4)	<0.001

a CAP, community-acquired pneumonia; HAP, hospital-acquired pneumonia; IQR, interquartile range; SBP, spontaneous bacterial peritonitis; VAP, ventilator-associated pneumonia.

b Regardless of susceptibility.

Of the requested specimens, 284 (65.4%) specimens were fluid, which included bronchoalveolar lavage (BAL) fluid (*n* = 97), abscess fluid (*n* = 48), cerebrospinal fluid (CSF) (*n* = 46), synovial fluid (*n* = 31), vitreous humor fluid (*n* = 23), pleural effusion (*n* = 21), pericardial effusion (*n* = 6), ascites (*n* = 5), blood from bone marrow (*n* = 5), and amniotic fluid (*n* = 2). The remaining 150 (34.6%) specimens were tissue (see Table S1 in the supplemental material). Of the specimens, 60 (14.6%) were positive by Gram staining, and 131 (30.2%) were positive by culturing. On 16S rRNA gene sequencing, 108 (24.9%) and 326 (75.1%) specimens gave positive and negative results, respectively. The overall median (IQR) turnaround time for 16S rRNA gene sequencing was 3 (2 to 10) days, with 11 (9 to 13) days in the 16S rRNA gene sequencing-positive group and 2 (1 to 4) days in the 16S rRNA gene sequencing-negative group. Comparisons of the characteristics between the 16S rRNA gene sequencing-positive and -negative groups are shown in Table 1. The proportions of males and the numbers of positive Gram stain and culture results were all higher in the 16S rRNA gene sequencing-positive group (*P* < 0.05).

Approximately two-thirds of patients in this study were receiving antibiotics regardless of susceptibility at the time of specimen collection. There was no significant difference in rates of 16S rRNA gene sequencing positivity regarding the receipt of antibiotics at the time of specimen collection: 75.0% in the 16S rRNA gene sequencing-positive group and 65.3% in the 16S rRNA gene sequencing-negative group (*P* = 0.063). The overall median (IQR) number of days of antibiotic treatment prior to specimen collection was 8 (3 to 18) days, with 10 (5 to 21) days in the 16S rRNA gene sequencing-positive group and 7 (2 to 16) in the 16S rRNA gene sequencing-negative group (*P* = 0.027). The remaining patients in this study (20.3%) were not receiving antibiotics at the time of specimen collection but had recently received antibiotics, and 12.0% had never received antibiotics. There was no significant difference in the proportions of patients with recent antibiotic exposure between the 16S rRNA gene sequencing-positive and -negative groups (Table 1): 6.7% and 13.6% of patients were exposed to antibiotics within 14 and 90 days, respectively.

Regarding the specimen type, there was no difference in the specimen types (fluid or tissue) between the 16S rRNA gene sequencing-positive and -negative groups (*P* = 0.140). Of the fluid specimens, those with a volume of more than 1.5 mL (mostly BAL fluid or abscess fluid) had a higher proportion of 16S rRNA gene sequencing-positive results than those with a volume of ≤1.5 mL, which were mostly vitreous humor, synovial, or cerebrospinal fluid. However, this was not a statistically significant difference (fluid volume of >1.5 mL, 31.6%, versus fluid volume of ≤1.5 mL, 24.1%; *P* = 0.166). In the abscess specimens, the proportion of 16S rRNA gene sequencing-positive results was higher, but statistical significance was not reached in the fluid with a volume of >1.5 mL compared with the fluid with a volume of ≤1.5 mL (75.0% versus 50.0%; *P* = 0.179). In contrast, in BAL fluid, the proportion of 16S rRNA gene sequencing-positive results was significantly lower in the fluid with a volume of >1.5 mL than in the fluid with a volume of ≤1.5 mL (fluid volume of >1.5 mL, 48.4%, versus fluid volume of ≤1.5 mL, 81.0%; *P* = 0.022). In tissue specimens, the proportions of 16S rRNA gene sequencing-positive and -negative results were similar (21/103, 20.4%, versus 10/47, 21.3%; *P* = 0.901), and this did not differ between the types of provisional diagnoses (*P* ≥ 0.05 for all provisional diagnoses).

### 16S rRNA gene PCR and sequencing results.

Regarding the final diagnosis, 253 (58.3%) specimens were collected from patients whose final diagnosis was a bacterial infection, whereas 181 (41.7%) specimens were from patients whose final diagnosis was a nonbacterial infection. Of the nonbacterial infection cases, 53 (29.3%) were caused by fungal infection or viral infection.

Table 2 shows the 16S rRNA gene sequencing results in this study. The 16S rRNA gene sequencing gave a positive result for 97 specimens (38.3% [97/253] sensitivity) of bacterial infection cases (22.4% of the total specimens) and a negative result for 170 specimens (93.9% [170/181] specificity) of nonbacterial infection cases (39.2% of the total specimens). The 16S rRNA gene sequencing indicated a suspected mixed organism infection in 12 specimens (2.8% of the total specimens). In the 16S rRNA gene sequencing-negative group, 40 (12.3%) specimens gave a positive amplification product for the 16S rRNA gene, but a causative bacterial pathogen failed to be identified by sequencing (9.2% of the total specimens).

**TABLE 2:** Comparison of 16S rRNA sequencing results categorized by bacterial infection and nonbacterial infection

16S rRNA sequencing result^*a*^	No. (%) of infections		
	Bacterial	Nonbacterial	Total
Positive	97 (22.4)	11 (2.5)	108
Negative	156 (35.9)	170 (39.2)	326
Total	253	181	434

a Positive included a result identifiable to bacterial genus or species and a result that reported mixed organisms. Negative included a result where the PCR product was not detected and where the 16S rRNA gene was detected but failed to identify bacteria by sequencing.

Among the bacterial infection cases, 16S rRNA gene sequencing gave a negative result in 156 (61.7%) specimens (35.9% of the total specimens) (Table 2). The yields for 16S rRNA gene sequencing and bacterial culture among bacterial infection cases are shown in Table 3. Of these cases, 82 (32.4%) specimens were concordantly positive by 16S rRNA gene sequencing and culture, and 130 (51.4%) specimens were concordantly negative by 16S rRNA gene sequencing and culture. The agreement between bacterial culture and 16S rRNA gene sequencing was 83.8% (κ coefficient, 0.664; *P* < 0.001). The proportion of 16S rRNA gene sequencing-positive specimens was 10.3% (15/145) in the culture-negative bacterial infection cases (5.9% of the total bacterial infection specimens), whereas the proportion of 16S rRNA gene sequencing-negative specimens was 24.1% (26/108) in the culture-positive bacterial infection cases (10.3% of the total bacterial infection specimens).

**TABLE 3:** Comparison of bacterial culture and 16S rRNA gene sequencing among bacterial infection cases

16S rRNA sequencing result^*a*^	No. (%) of cases		
	Culture positive	Culture negative	Total
Positive	82 (32.4)	15 (5.9)	97
Negative	26 (10.3)	130 (51.4)	156
Total	108	145	253

a Positive included an identifiable result to bacterial genus or species and a result that reported mixed organisms. Negative included a result where the PCR product was not detected and where the 16S rRNA gene was detected but failed to identify bacteria by sequencing.

Table 4 shows the bacterial culture and 16S rRNA gene sequencing results based on a provisional diagnosis in patients with bacterial infection as the cause for the final diagnosis. In skin and soft tissue infection cases, abscess specimens had a higher proportion of 16S rRNA gene sequencing-positive and culture-negative results than tissue specimens (44.4% versus 0.0%). In CAP/HAP/VAP cases, most specimens gave a positive culture result, only 1/14 (7.1%) specimens gave a 16S rRNA gene sequencing-positive and culture-negative result, and the causative agent was reported as Pseudomonas aeruginosa. The patient was intubated the next day, and tracheal suction culture results showed intermediate susceptibility for the organism. The patient received piperacillin-tazobactam for 9 days before BAL fluid collection.

**TABLE 4:** Bacterial culture and 16S rRNA gene sequencing results based on provisional diagnoses among patients with a final diagnosis caused by bacterial infection^*a*^

Provisional diagnosis	Specimen type (*n*)	No. (%) of results/total (*n* = 253)		
		Culture positive and sequencing positive (*n* = 108)	Culture negative and sequencing positive (*n* = 145)	Sequencing positive
HAP/VAP	BAL fluid (24)	16/21 (76.2)	0/3 (0.0)	16/24 (66.7)
CAP	BAL fluid (24)	13/13 (100)	1/11 (9.1)	14/24 (58.3)
Lung abscess	BAL fluid (2)	1/1 (100)	0/1 (0.0)	1/2 (50.0)
	Tissue (1)	0/0 (0.0)	0/1 (0.0)	0/1 (0.0)
Parapneumonic effusion	Pleural effusion (11)	1/2 (50.0)	0/9 (0.0)	1/11 (9.1)
Skin and soft tissue infection	Abscess (16)	6/7 (85.7)	4/9 (44.4)	10/16 (62.5)
	Tissue (23)	9/12 (75.0)	0/11 (0.0)	9/23 (39.1)
Septic arthritis	Synovial fluid (18)	3/6 (50.0)	1/12 (8.3)	4/18 (22.2)
	Tissue (15)	4/6 (66.7)	2/9 (22.2)	6/15 (40.0)
Prosthetic joint infection	Synovial fluid (2)	0/0 (0.0)	0/2 (0.0)	0/2 (0.0)
	Tissue (10)	3/6 (50.0)	0/4 (0.0)	3/10 (30.0)
Native vertebral osteomyelitis	Abscess (4)	0/0 (0.0)	0/4 (0.0)	0/4 (0.0)
	Bone (4)	1/1 (100)	1/3 (33.3)	2/4 (50.0)
	Tissue (10)	0/0 (0.0)	1/10 (10.0)	1/10 (10.0)
Osteomyelitis	Bone (9)	4/6 (66.7)	0/3 (0.0)	4/9 (44.4)
	Tissue (6)	2/4 (50.0)	0/2 (0.0)	2/6 (33.3)
Postoperative meningitis	CSF (8)	1/2 (50.0)	0/6 (0.0)	1/8 (12.5)
Brain abscess/epidural abscess	Abscess (1)	0/0 (0.0)	1/1 (100)	1/1 (100)
	Tissue (2)	1/1 (100)	0/1 (0.0)	1/2 (50.0)
Meningitis	CSF (6)	0/0 (0.0)	0/6 (0.0)	0/6 (0.0)
Uveitis/endophthalmitis/vitritis (eye)	Vitreous humor (18)	4/5 (80.0)	1/13 (7.7)	5/18 (27.8)
	Tissue (1)	0/0 (0.0)	0/1 (0.0)	0/1 (0.0)
Infective endocarditis	Tissue (2)	0/1 (0.0)	0/1 (0.0)	0/2 (0.0)
Peri/myocarditis	Pericardial effusion (2)	0/0 (0.0)	0/2 (0.0)	0/2 (0.0)
Lymphadenopathy	Abscess (1)	1/1 (100)	0/0 (0.0)	1/1 (100)
	Tissue (2)	0/0 (0.0)	0/2 (0.0)	0/2 (0.0)
Intra-abdominal collection	Fluid (8)	2/2 (100)	1/6 (16.7)	3/8 (37.5)
	Tissue (2)	1/2 (50.0)	0/0 (0.0)	1/2 (50.0)
Liver abscess	Abscess (7)	5/5 (100)	0/2 (0.0)	5/7 (71.4)
Renal abscess	Abscess (2)	0/0 (0.0)	2/2 (100)	2/2 (100)
Perinephric collection	Fluid (3)	2/2 (100)	0/1 (0.0)	2/3 (66.7)
Splenic abscess	Abscess (1)	0/0 (0.0)	0/1 (0.0)	0/1 (0.0)
SBP	Ascites (3)	0/0 (0.0)	0/3 (0.0)	0/3 (0.0)
Colitis	Tissue (1)	1/1 (100)	0/0 (0.0)	1/1 (100.0)
Chorioamnionitis	Amniotic fluid (2)	0/0 (0.0)	0/2 (0.0)	0/2 (0.0)
Bone marrow infection	Blood from BM (1)	0/0 (0.0)	0/1 (0.0)	0/1 (0.0)

a BAL, bronchoalveolar lavage; BM, bone marrow; CAP, community-acquired pneumonia; CSF, cerebrospinal fluid; HAP, hospital-acquired pneumonia; SBP, spontaneous bacterial peritonitis; VAP, ventilator-associated pneumonia.

Osteomyelitis cases were associated with the highest proportions of 16S rRNA gene sequencing-positive results among bone and joint infection cases (40.0%), followed by septic arthritis (31.3%), prosthetic joint infection (25.0%), and native vertebral osteomyelitis (16.7%) cases.

The provisional diagnoses in cases that were 16S rRNA gene sequencing-positive but culture-negative included skin and soft tissue infection (*n* = 4), septic arthritis (*n* = 3), native vertebral osteomyelitis (*n* = 2), renal abscess (*n* = 2), community-acquired pneumonia (*n* = 1), brain abscess (*n* = 1), endophthalmitis (*n* = 1), and intra-abdominal collection/abscess (*n* = 1) (see Fig. S1 and S2 in the supplemental material).

### Impact on antimicrobial management.

Of the 434 specimens, 15 specimens were 16S rRNA gene sequencing-positive and culture-negative and only 10 specimens (2.3% of the total specimens) from eight cases had an impact on antimicrobial management (6.9% of the culture-negative bacterial infection cases). The impact on microbial management in all of these cases was the continuation of antibiotics. The details of these cases are shown in Table 5 (Table S2). Three cases that were 16S rRNA gene sequencing-positive and culture-negative resulted in an identified pathogen, but this failed to have an impact on antimicrobial management. The identified pathogens in these three cases were as follows: (i) Fusobacterium nucleatum in a brain abscess, where the result was returned after the patient had died; (ii) Enterococcus faecalis in native vertebral osteomyelitis, where the result was returned after the patient had died from infected wet gangrene; and (iii) Klebsiella pneumoniae in an intra-abdominal collection/abscess, where the antibiotic course was completed before the result was reported. The remaining two 16S rRNA gene sequencing-positive and culture-negative specimens did not result in antimicrobial adjustment because of the long turnaround time (range of 8 to 13 days) for the sequencing results, and the identification of the bacterial culture from the same site as the previous investigation was reported before the sequencing results were returned.

**TABLE 5:** Details of cases with 16S rRNA sequencing-positive and culture-negative specimens

Clinical diagnosis (*n* = 15)	Specimen type (specimen no.)	Impact on diagnosis/treatment	16S rRNA sequencing identification	Antibiotic use at specimen collection	Turnaround time (days)	Final diagnosis
Positive impact on antimicrobial management (*n* = 10)						
Renal abscess	Abscess (60)^*a*^	1st identify pathogen/continuation of antibiotic	Klebsiella pneumoniae	25 days of meropenem (susceptible)	9	K. pneumoniae-infected renal cyst; urine culture positive (K. pneumoniae); blood culture negative
	Abscess (71)^*a*^	2nd confirm current pathogen (clinical worsening)/continuation of antibiotic	Klebsiella pneumoniae	29 days of meropenem (susceptible)	12	K. pneumoniae-infected renal cyst; urine culture positive (K. pneumoniae); blood culture negative

Skin and soft tissue infection	Abscess (124)	Confirm diagnosis/continuation of antibiotic	Pseudomonas aeruginosa	22 days of meropenem (susceptible)	12	P. aeruginosa intramuscular abscess; blood culture positive (P. aeruginosa) last 22 days
	Abscess (270)^*b*^	Confirm diagnosis/continuation of antibiotic	Streptococcus gallolyticus subsp. *pasteurianus*	10 days of vancomycin and piperacillin-tazobactam (susceptible)	12	S. gallolyticus subsp. *pasteurianus* myositis; blood culture positive (S. gallolyticus subsp. *pasteurianus*) last 9 days
	Abscess (284)^*b*^	Confirm diagnosis/continuation of antibiotic	Streptococcus gallolyticus subsp. *pasteurianus*	24 days penicillin G (susceptible)	13	*S. gallolyticus* subsp. *pasteurianus*-infected hematoma; blood culture positive (*S. gallolyticus* subsp. *pasteurianus*) last 24 days
	Abscess (313)	Confirm diagnosis/continuation of antibiotic	Klebsiella pneumoniae	25 days of ceftriaxone (susceptible)	12	K. pneumoniae myositis; blood culture positive (Micrococcus luteus) last 16 days

Native vertebral osteomyelitis	Bone (249)	Confirm diagnosis/continuation of antibiotic	Streptococcus gallolyticus subsp. *pasteurianus*	16 days of penicillin G (susceptible)	11	S. gallolyticus subsp. *pasteurianus* spondylodiscitis; blood culture positive (S. gallolyticus subsp. *pasteurianus*) last 16 days

Septic arthritis	Synovial tissue (235)	Confirm diagnosis (worsening septic arthritis)/continuation of antibiotic	Streptococcus agalactiae	31 days of penicillin G (susceptible)	7	S. agalactiae septic arthritis; blood and synovial fluid culture positive (S. agalactiae) last 30 days
	Tissue (227)	Confirm diagnosis/continuation of antibiotic	Streptococcus pyogenes	27 days of amoxicillin-clavulanate (susceptible)	10	Streptococcus pyogenes septic arthritis; blood culture negative

Endophthalmitis	Vitreous humor (28)	Confirm diagnosis/continuation of antibiotic	Klebsiella pneumoniae	3 days of meropenem (susceptible)	12	K. pneumoniae endophthalmitis; blood culture positive (K. pneumoniae) last 3 days
No impact on antimicrobial management (*n* = 5)						
Native vertebral osteomyelitis	Tissue (91)	Identify pathogen/long turnaround time (patient expired)	Enterococcus faecalis	12 days of ceftriaxone (nonsusceptible)	12	Enterococcus faecalis native vertebral osteomyelitis; blood culture positive (K. pneumoniae) last 12 days
Brain abscess	Abscess (299)	Identify pathogen/long turnaround time (patient expired)	Fusobacterium nucleatum	28 days of ceftriaxone and metronidazole (susceptible)	8	Fusobacterium nucleatum brain abscess; blood culture negative
Intra-abdominal collection/abscess	Abscess (348)	Identify pathogen/long turnaround time; complete antibiotic 8 days before result reported	Klebsiella pneumoniae	5 days of ceftriaxone (susceptible)	10	Acute cholecystitis with cholangitis; blood culture negative
Community-acquired pneumonia	Bronchoalveolar lavage fluid (288)	Long turnaround time (positive tracheal suction [*Pseudomonas aeruginosa*] next 4 days)	Pseudomonas aeruginosa	9 days of piperacillin-tazobactam (intermediate susceptible)	12	P. aeruginosa community-acquired pneumonia; blood culture negative
Septic arthritis	Synovial fluid (355)	Confirm diagnosis/long turnaround time (previous synovial culture reported 1 day after specimen collection)	Streptococcus dysgalactiae	2 days of penicillin G (susceptible)	11	Streptococcus dysgalactiae septic arthritis; blood culture positive (S. dysgalactiae) last 2 days

a Specimens 60 and 71 were collected from the same patient.

b Specimens 270 and 284 were collected from the same patient.

Discordance between the pathogen identified by 16S rRNA gene sequencing and that identified by culturing was observed in five cases. Four specimens were positive for anaerobic bacteria, and one specimen was positive for *Mycoplasma* spp., whereas the culture results all indicated aerobic bacteria (Table S3). Among these specimens, the 16S rRNA gene sequencing results were considered to have a clinical correlation in three cases (Bacteroides fragilis, Achromobacter xylosoxidans, and Prevotella oris). The results from the other two cases were considered to indicate colonizing bacteria (Bacteroides fragilis and *Mycoplasma* spp.), as determined by the final diagnosis from the attending physician.

## DISCUSSION

We showed the clinical value of 16S rRNA gene sequencing in a real-world situation with no other restrictions on testing. The 16S rRNA gene sequencing identified bacteria in 38.3% of bacterial infection cases. However, only in the case of 10 specimens (2.3%) did this identification have an impact on antimicrobial management, resulting in the continuation of antibiotic treatment in all cases.

The overall positive rate for 16S rRNA gene sequencing in this study (24.9%) was similar to that of previous studies. Overall positive rates of 17.1% and 20.8% were reported by Fida et al. (16) and Teoh et al. (17), respectively. The proportion of specimens that were 16S rRNA gene sequencing-positive and culture-negative was also concordant between our study and previous studies: 10.3% versus 11.1% and 10.0%, respectively. Our study showed that a positive result was obtained for 16S rRNA gene sequencing in 78.3% of clinical specimens with a positive Gram stain. This result was consistent with a previous report that specimens with a positive Gram stain were 12 times more likely to have a positive 16S rRNA gene sequencing result than those with a negative Gram stain (16). The proportion of positive 16S rRNA gene sequencing results in patients currently on antibiotics remained high (75.0%). This implied that 16S rRNA gene sequencing could identify pathogens from patients who have been treated with antibiotics, corresponding with previous studies (10, 13, 18). This may be explained by the ability of PCR to detect both viable and nonviable bacteria or may be indicative of nonsusceptibility to current antibiotics.

The concordance between bacterial identification from blood culture and 16S rRNA gene sequencing results from an infected site in this study was high. Of the eight cases (nine specimens) with positive blood cultures, the 16S rRNA sequencing results were concordant in six cases (75.0%), especially for abscesses and bone and joint infections. This finding indicates that 16S rRNA gene sequencing of specimens from an infected site in patients with bacteremia might not be necessary if a clinical response is observed.

The most common provisional diagnosis in this study was skin and soft tissue infection. The results of this study demonstrated that the results obtained for all abscess specimens that were culture negative and 16S rRNA gene sequencing test positive had an impact on antimicrobial management. In contrast, for cases of CAP/HAP/VAP, which was the second most common provisional diagnosis in this study, 16S rRNA gene sequencing results had no impact on antimicrobial management. Previous studies showed the benefit of 16S rRNA gene sequencing of cardiovascular and bone and joint infection specimens in identifying bacterial pathogens and aiding the management of antimicrobial therapy (16, 19, 20). However, in this study, the results from only 3/78 (3.8%) specimens from bone and joint infections had an impact on antimicrobial management. Our study involved only a limited number of cases of cardiovascular infection, and therefore, the impact of analysis of such specimens on antimicrobial management could not be determined from our study.

The impact of 16S rRNA gene sequencing on antimicrobial management in this study was lower than reported in previous studies, which ranged from 2.2% to 41.6%, depending on the inclusion criteria and the definition for the impact on antimicrobial management (16, 17, 21–24). The limited impact of 16S rRNA gene sequencing in this study may be because (i) nearly half of the tests were requested in cases with a final diagnosis relating to nonbacterial infectious cases, (ii) a small proportion of specimens gave a positive result with 16S rRNA gene sequencing but a negative result on culturing (10.3% of the specimens), (iii) the long turnaround time required to obtain the sequencing results meant that empirical treatment/switching to oral antibiotics was completed before the results were reported (although previous studies reported a similar turnaround time of 6 to 13 days) (17, 22, 24, 25), (iv) the relatively low sensitivity of 16S rRNA gene sequencing in detecting bacterial pathogens led to physicians waiting for culture results when the sequencing yielded negative results, and (v) this study included all types of specimens, which may have resulted in various degrees of positive diagnoses and variations in antimicrobial management. The impact seemed to be lower when specimens were from nonspecific sites (including specimens from all provisional diagnoses), and two previous studies showed impacts of 2.2% and 6.4% (16, 17) compared with higher values for specific diseases such as intracranial infections (41.7%) (22) and infective endocarditis (31%) (14).

Previous studies reported 95.7% positive 16S rRNA gene pyrosequencing results among BAL fluid specimens from symptomatic and asymptomatic lung transplant recipients (26). Pyrosequencing identified a greater percentage of bacteria than culturing. However, 16S rRNA gene sequencing from BAL fluid in our study, using Sanger sequencing, identified only one case (6.7%) in the culture-negative group and did not have an impact on antimicrobial management. In cases with bacterial infection, bacteria were identified by culturing in 70.0% of BAL fluid specimens. The high number of positive cultures was consistent with previous studies (27, 28).

The proportion of 16S rRNA gene sequencing-positive specimens was higher when the fluid volume was >1.5 mL than with a fluid volume of ≤1.5 mL, but this difference was not statistically significant. This was in contrast to BAL fluid, for which a volume of ≤1.5 mL yielded a significant number of 16S rRNA gene sequencing-positive results. This might be explained by the fact that BAL fluid is collected by saline instillation of a subsegment of the lung, which results in dilution of the fluid, whereas other fluids are not diluted. Therefore, in fluid specimens other than BAL fluid, a large amount of fluid is preferred.

Although 16S rRNA gene sequencing had an impact on antimicrobial management in some cases, the result of 16S rRNA gene sequencing from a clinical specimen should be interpreted with caution. A positive sequencing result should take into consideration the possibility of colonization, a mixed infection (especially with anaerobic bacteria), and contamination. Equally, a negative sequencing result might not exclude bacterial infection. Discordance between 16S rRNA gene sequencing results and cultured organisms occurred in polymicrobial infection/colonization. Furthermore, 16S rRNA gene sequencing does not provide data on antibiotic susceptibility.

The strengths of this study were that we obtained a large number of clinical specimens in cases with various provisional diagnoses in a setting with no test ordering restrictions. There were no missing data as a result of the prospective design of the study. All cases had a final diagnosis based on the microbiological results and the clinical response to antimicrobial management. We accepted the limitations that a large proportion of specimens were obtained from cases with nonbacterial infections and a small number of clinical specimens were available for some diseases. The data were collected in a single hospital center representing a limited geographic region. Additionally, comparison of 16S rRNA gene sequencing to culture methods in this setting was not an equal comparison because the long turnaround time associated with gene sequencing inherently led to delayed treatment decisions.

In summary, testing by 16S rRNA gene sequencing has improved clinical management in selected cases but cannot replace conventional culture. The overall diagnostic yield with 16S rRNA gene sequencing in bacterial infection cases was fair. The yields for 16S rRNA gene sequencing were highest in specimens from skin and soft tissue infection and pneumonia. Sequencing of specimens obtained from abscesses was the most likely to be beneficial, whereas sequencing of BAL fluid specimens had the lowest impact on antimicrobial management as a result of the long turnaround time to obtain sequencing results. In our study in Thailand, where the turnaround time for sequencing results was prolonged, we suggest performing 16S rRNA gene sequencing in chronic cases in which bacterial infection is suspected and conventional culture was negative, in infection cases where the suspected pathogen is difficult to culture or unculturable, or in cases that did not respond to initial treatment. Additionally, to improve the application of this test, restrictions on test requests should be permitted only in cases involving the above-mentioned scenarios and when bacteria are not isolatable from culture.

## MATERIALS AND METHODS

### Recruitment.

A prospective study was conducted among adult (age of ≥18 years) inpatients for whom the attending physician requested 16S rRNA gene sequencing from a direct clinical specimen at Ramathibodi Hospital (a 1,300-bed university hospital in Bangkok, Thailand) between January and December 2021. Specimens that were requested for 16S rRNA gene sequencing without a corresponding bacterial culture, or as a repeated order for a follow-up, were excluded. The study was approved by the Human Research Ethics Committee, Faculty of Medicine Ramathibodi Hospital, Mahidol University (approval no. COA.MURA2020/1962). All participants provided written informed consent. There were no restrictions on ordering 16S rRNA gene sequencing at the time of the study. Clinical and demographic data were collected from the medical records, including age, sex, immune status, provisional diagnosis, time of antibiotic initiation, previous antibiotic exposure, adjustment of antibiotic upon a 16S rRNA gene sequencing result, and final diagnosis. Microbiological data, such as specimen type, quantity, Gram stain, culture result, and turnaround time, were compiled. The investigators were not involved in the clinical decisions or management during antimicrobial treatment because of concerns regarding potential bias that may originate from interactions between investigators and the attending physician. The attending physician was only contacted after antimicrobial treatment when data from medical records were incomplete.

### Definition.

The 16S rRNA gene sequencing-positive group included the results that identified bacterial genus/species or reported/suspected a mixed organism infection (29); the 16S rRNA gene sequencing-negative group was defined as the sequencing results that were unable to identify bacterial genus/species regardless of 16S rRNA PCR target detection.

16S rRNA gene sequencing was defined as having an impact on antimicrobial management when the result of 16S rRNA gene sequencing resulted in a continued/extended, adjusted, or withheld antibiotic coinciding with the date from which the 16S rRNA gene sequencing result was reported until antibiotic completion.

Bacterial infections included those caused by aerobes, anaerobes, spirochetes, the Mycobacterium tuberculosis complex, and nontuberculous mycobacteria. Bacterial infection as a cause of disease was concluded according to the final diagnosis determined by an attending physician based on the patient’s clinical condition, and/or microbiological and/or molecular results, and the response to antimicrobial treatment.

A nonbacterial cause of infection was concluded according to the final diagnosis determined by an attending physician. Examples of nonbacterial infection conditions included viral and fungal infections, as well as autoimmune disease, inflammatory disease, malignancy, and an adverse drug event.

### Bacterial identification.

All specimens were processed at the Microbiology Laboratory, Department of Pathology, Faculty of Medicine Ramathibodi Hospital, Mahidol University, Bangkok, Thailand. Bacterial isolates were identified using MALDI-TOF MS (BD, Franklin Lakes, NJ, USA). In the case of inconclusive identification by MALDI-TOF MS, the isolates were further identified by 16S rRNA gene sequencing. Antimicrobial susceptibility testing was performed and interpreted according to the guidelines of the Clinical and Laboratory Standards Institute (CLSI) (30).

### 16S rRNA gene sequencing and bacterial species identification.

For fluid specimens (e.g., bronchoalveolar lavage [BAL] fluid and abscess fluid), 1.5 mL of the fluid specimen was collected and centrifuged (15,000 × *g*, 5 min). Then, 1.1 mL of clear supernatant was removed and the remaining 400 μL of fluid was used for DNA extraction. If the volume of the specimen was less than 1.5 mL, 400 μL of the fluid was used for DNA extraction without centrifugation. For bone marrow samples taken from blood culture bottles, 2 mL of sample was washed twice with DNA/RNA-free distilled water and centrifuged at 15,000 × *g*. After the second wash, ~1.6 mL of supernatant was removed, and the pellet was resuspended in the remaining supernatant, prior to further processing. For the bone marrow sample in the EDTA tube, 2 mL of sample was centrifuged, and ~0.4 mL of the pellet was used for DNA extraction.

For tissue specimens, ~10-mg pieces of tissue were mixed with 400 μL of tissue lysis buffer and 20 μL of proteinase K (20 mg/mL) (Precision System Science, Chiba, Japan) before being incubated overnight at 56°C. After incubation, specimens were centrifuged and 400 μL of the clear supernatant was used for DNA extraction. All specimens were extracted using a magLEAD-12gC extraction robot (Precision System Science).

A conventional PCR was performed to amplify the V1-to-V3 hypervariable regions of the 16S rRNA gene. The primers used in the PCR were as follows: forward primer 27F (5′-AGA GTT TGA TCM TGG CTC AG-3′) and reverse primer 519R (5′-GWA TTA CCG CGG CKG CTG-3′) (Bioneer, Daejeon, the Republic of Korea) (31). Amplified PCR products were stored at 4°C and then sent via air transport from Thailand to the Bioneer Corporation in the Republic of Korea, at least twice a week, for Sanger sequencing on a 3730xl DNA analyzer (Applied Biosystems, Waltham, MA, USA). Obtained sequences were aligned using BLAST for bacterial species identification (32).

### Statistical analysis.

The mean ± standard deviation or median (IQR) was used for continuous variables, whereas the frequency and percentage were used for categorical variables. The Mann-Whitney U test or *t* test was used to compare continuous variables. Fisher’s exact test or the chi-square test was used to compare categorical variables. A *P* value of <0.05 was considered statistically significant. The sensitivity and specificity of 16S rRNA sequencing were calculated to assess diagnostic performance using the final diagnosis as a “gold standard.” The κ coefficient was used to estimate the agreement between the results of 16S rRNA gene sequencing and conventional culture. All statistical analyses were performed using SPSS Statistics 25.0 (IBM, Armonk, NY, USA).
