# Supplementary material for: Diagnostic Yield and Impact on Antimicrobial Management of 16S rRNA Testing of Clinical Specimens
Source: Microbiol Spectr. 2022 Nov 14;10(6):e02094-22. doi: 10.1128/spectrum.02094-22 (PMC9769669; doi:10.1128/spectrum.02094-22)
Supplement: Supplemental file 1 — Fig. S1 and S2 and Tables S1 to S3. Download spectrum.02094-22-s0001.pdf, PDF file, 0.3 MB [file spectrum.02094-22-s0001.pdf]

## Supplementary Figures

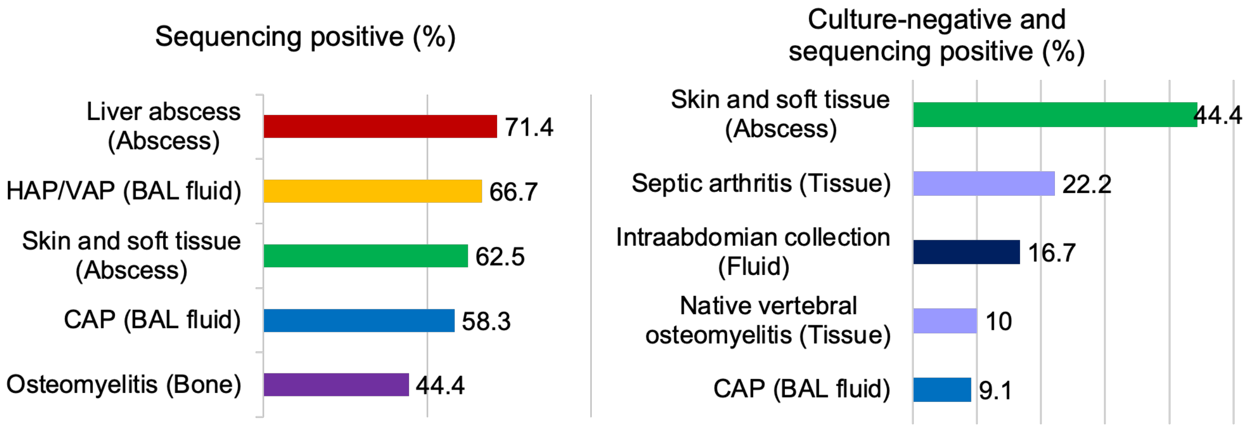


**Fig. S1** Ranking proportion of bacterial culture and 16s rRNA gene sequencing results (exclude N <5)

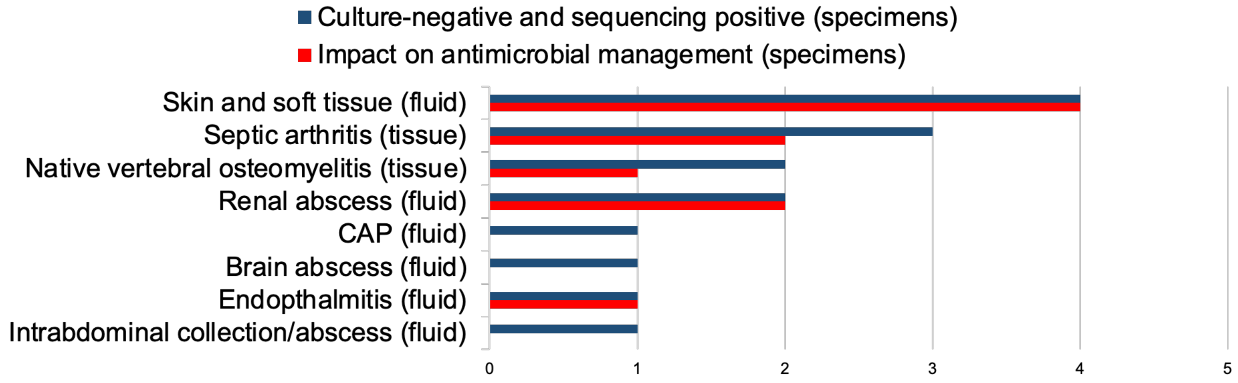


**Fig. S2** Comparison between the number of culture-negative and sequencing positive specimens and the number of impacts on antimicrobial management

**Supplementary Tables**

**Table S1.** Characteristic of the specimens in this study

| **Specimen** | **All (n=434)** | **16s rRNA gene sequencing positive (n=108)** | **16s rRNA gene sequencing negative (n=326)** | **P-value** |
|---|---|---|---|---|
| Fluid, n (%) | 284 (65.4) | 77 (71.3) | 207 (63.5) | 0.140 |
| Fluid volume | | | | |
| ≤1.5 mL | 170 (59.9) | 41/77 (53.2) | 129/207 (62.3) | 0.166 |
| >1.5 mL | 114 (40.1) | 36/77 (46.8) | 78/207 (37.7) | |
| Fluid type | | | | <0.01 |
| Bronchoalveolar lavage | 97 | 42 (43.3) | 55 (56.7) | |
| Abscess | 48 | 23 (47.9) | 25 (52.1) | |
| Cerebrospinal fluid | 46 | 1 (2.2) | 45 (97.8) | |
| Synovial fluid | 31 | 4 (12.9) | 27 (87.1) | |
| Vitreous humor | 23 | 5 (21.7) | 18 (78.3) | |
| Pleural effusion | 21 | 1 (4.8) | 20 (95.2) | |
| Pericardial effusion | 6 | 0 (0) | 6 (100) | |
| Ascites | 5 | 1 (20.0) | 4 (80.0) | |
| Bone marrow aspiration | 5 | 0 (0) | 5 (100) | |
| Amniotic fluid | 2 | 0 (0) | 2 (100) | |
| Tissue, n (%) | 150 (34.6) | 31 (28.7) | 119 (36.5) | 0.140 |
| Tissue volume | | | | |
| Tissue ≤10 mg | 103 (68.7) | 21/31 (67.7) | 82/119 (68.9) | 0.901 |
| Tissue >10 mg | 47 (31.3) | 10/31 (32.3) | 37/119 (31.1) | |
| Tissue type | | | | 0.485 |
| Soft tissue | 127 | 25 (19.7) | 102 (80.3) | |
| Bone | 23 | 6 (26.1) | 17 (73.9) | |

**Table S2.** Clinical course of the eight cases that the investigations showed 16s rRNA gene sequencing positive/culture-negative results and had the impact on antimicrobial management

| Specimen number | Clinical course |
| --- | --- |
| 60 and 71 | An 89-year-old man with hypertension, atrial fibrillation, chronic kidney disease, and benign prostatic hyperplasia presented with fever, cloudy urine and right frank pain for three days. Physical examination revealed tenderness at the right costovertebral angle. Urinalysis showed pyuria. He was diagnosed with acute pyelonephritis, and meropenem was given as an empirical antibiotic as he was recently discharged from the hospital. Hemoculture and urine culture later grew ESBL-producing *Klebsiella pneumoniae*. The antibiotic was then narrowed to ertapenem. Ultrasonography of the kidney demonstrated a 4-cm complex cyst at the kidney. Later, the patient developed fever and septic shock during receiving ertapenem. Meropenem was then given to board antibiotic spectrum. Computed tomography (CT) scan of the abdomen was performed and demonstrated several cysts in both kidneys with wall enhancement and increase in size. Fluid aspiration from a renal cyst was performed. Pus culture grew no organism. The 16s rRNA gene sequencing result later identified *K. pneumoniae* (specimen #60). Meropenem was continued. The patient still had fever and occasionally hypotensive. Fluid aspiration was, again, performed, and grew no organism. The 16s rRNA gene sequencing result, again, identified *K. pneumoniae* (specimen #71). Meropenem was continued. Because the presence of numerous cysts, and the patient's clinical was not improved, decortication of right renal cysts was performed. Tissue and pus cultures grew no organism, and the 16s rRNA gene sequencing identified no organism. Meropenem was continued for a total duration of 90 days. |
| 124 | A 32-year-old man with relapsed acute lymphoblastic leukemia admitted to the hospital due to fever with gum pain and bleeding. Physical examination further revealed redness and swelling at the left leg. Laboratory investigations showed neutropenia, and Meropenem was given as an empirical antibiotic. Hemoculture later grew *Pseudomonas aeruginosa*. The antibiotic was then narrow to piperacillin/tazobactam. Later, the patient had pain on the left leg and increase redness on the lesion. Ultrasonography of the left leg demonstrated a heterogeneous hypoechoic lesion in the intramuscular of the left leg. The antibiotic was switch to meropenem. The lesion was then aspirated, and fluid was sent for investigations. Fluid culture grew no organism. Later, the 16s rRNA result reported *P. aeruginosa.* The patient's clinical course was then improved and meropenem was then discontinued. |
| 270 and 284 | A 39-year-old woman diagnosed with SLE with lupus nephritis, anti-phospholipid syndrome, pulmonary hypertension, and refractory ITP post |

| | |
|---|---|
| | splenectomy receiving immunosuppressants presented to the hospital with fever for two days prior to admission. The patient also had dyspnea. She was recently discharged from the hospital approximately two weeks ago due to *Streptococcus gallolyticus* Subsp. *pasteurianus* septicemia. She received susceptible antibiotics for a total duration of 14 days. Physical examination on this admission revealed fever, fine crackles both lower lungs, bilateral leg edema, loud P2, but no murmur. Chest film showed opacity and she also had cytomegalovirus (CMV) viremia. She was diagnosed with CMV pneumonia and received ganciclovir. Fever gradually subsided. On the third week of admission, she had high-grade fever and left thigh tenderness. Empirical antibiotics (vancomycin and piperacillin/tazobactam) were given. CT scan of the left thigh demonstrated a rim enhancing hypodense lesion along the left mid to lower thigh. Findings were probably fluid collection and/or resolving hematoma. Hemoculture grew *S. gallolyticus* Subsp. *pasteurianus*. Aspiration of the collection was performed 10 days after empirical antibiotic therapy. The aspiration revealed pus content, which pus culture grew no organism. The antibiotic was narrowed to penicillin G. The 16s rRNA gene sequencing result later revealed *S. gallolyticus* Subsp. *pasteurianus* (specimen #270). Antibiotic was therefore continued for a plan duration of one month. During the treatment, the patient still had low grade fever and left thigh pain, CT scan of the left thigh was then performed at three weeks after the treatment and showed increased size of a large retroperitoneal hematoma at the left posterior pararenal space with extension to left psoas muscle and pelvic cavity. Aspiration was again performed to rule out superimposed infection. Blackish fluid was obtained. Culture of the fluid grew no organism. Hemoculture taken five days before the aspiration also grew no organism. Because the diagnosis of infected hematoma could not be totally excluded, oral antibiotic (amoxycillin) was given after one-month course of intravenous antibiotics. The result of 16s rRNA sequencing later showed *S. gallolyticus* Subsp. *pasteurianus* (specimen #284). Oral antibiotic was then continued for a total duration of one month. |
| 313 | A 51-year-old man with diabetes mellitus and B-cell acute lymphoblastic leukemia who had recently received chemotherapy presented to the hospital with increased left leg pain and swelling for one week without fever. He was recently discharged from the hospital for one week due to a left leg abscess caused by *Klebsiella pneumoniae* and was receiving levofloxacin. Physical examination showed left leg swelling, warm, mild tenderness with no erythema. He was given piperacillin/tazobactam as an empirical antibiotic for a provisional diagnosis of soft tissue infection. Ultrasonography of the left leg showed new development of the large intramuscular collection. Hemoculture grew *Micrococcus luteus*, which is considered a contamination. After consultation with infectious disease team, piperacillin/tazobactam was narrowed to ceftriaxone, and open |

| | |
|---|---|
| | drainage was done on the following week; anchovy sauce-like pus at the amount of 300 mL was revealed intraoperatively. Pus culture grew no organism. After clinical improvement, the antibiotic was switched to oral levofloxacin. The 16s rRNA gene sequencing result later identified *K. pneumoniae*. Oral levofloxacin was continued for a total of two weeks after an adequate drainage. |
| 249 | A 70-year-old man presented to the hospital with low back pain for two weeks. Magnetic resonance imaging (MRI) revealed spondylodiscitis at L3-L5 with multiple right psoas abscesses. Piperacillin/tazobactam was given as an empirical antibiotic. The antibiotic was narrowed to penicillin G after hemoculture grew *Streptococcus gallolyticus*. Laminectomy and drainage of the abscess were done two weeks after the receiving antibiotic treatment. Bone culture grew no organism. The 16s rRNA gene sequencing result later showed *S. gallolyticus*. Penicillin G was continued. Because the patient had multiple hospital-acquired infections and drug-induced agranulocytosis, antibiotics were later adjusted based on subsequent infections. Unfortunately, the patient died after three months of hospitalization. |
| 235 | A 70-year-old man with his first diagnosis of diabetes mellitus (HbA1c 13.9%) presented to the hospital with right knee pain for five days. The right knee arthrocentesis was done, and gram-positive cocci in chain were found from a Gram stain. Ampicillin was given as an empirical antibiotic and narrowed to penicillin G after hemoculture and synovial fluid grew *Streptococcus agalactiae.* Multiple surgical debridements were done. After four weeks of the antibiotic, swollen and erythema of the right knee were observed. Surgical debridement was done. Tissue culture grew no organism. The 16s rRNA gene sequencing result later identified *S. agalactiae.* Penicillin G was continued. An MRI revealed multiple intramuscular rims enhancing fluid collection by up to 6 cm. Surgical debridement and drainage was done. The total duration of the antibiotic was seven weeks. |
| 227 | A 70-year-old man presented to the hospital with left knee erythema and tenderness. A left knee arthrocentesis was done and gram-positive cocci in chain was found. Ceftriaxone was empirically treated for three days and switched to levofloxacin after joint fluid culture grew *Streptococcus pyogenes*. Hemoculture grew no organism. The antibiotic was switched to amoxicillin/clavulanate for an additional nine days of treatment at an outpatient department without improvement. The patient was readmitted. Surgical debridement was done, and penicillin G was commenced. After tissue culture grew no organism, the patient was discharged with oral amoxicillin/clavulanate. On the next 10 days, the 16s rRNA gene sequencing result later showed *S. pyogenes*. Oral amoxicillin/clavulanate was continued for a total of four weeks with complete clinical response. |
| 28 | A 74-year-old man with coronary artery disease presented to the hospital with blurred left-eye vision for one day. Panuveitis was diagnosed. Intravitreal ceftazidime and intravenous meropenem were given as |

| | |
|---|---|
| | empirical antibiotics. The patient also had lung abscesses and neck abscesses. Hemoculture grew *Klebsiella pneumoniae,* vitreous fluid grew no organism. Antibiotic was narrowed to intravenous ceftriaxone. Twelve days later, the 16s rRNA gene sequencing result showed *K. pneumoniae,* ceftriaxone was continued. However, the patient developed a maculo-papular rash, and ceftriaxone was suspected a culprit drug. Thus, oral levofloxacin was prescribed for four weeks with complete clinical response. |

**Table S3.** Discordance between 16s rRNA sequencing and culturable organisms

| **Provisional diagnosis (specimen); final diagnosis** | **Culture** | **16s rRNA gene sequencing result** | **Reason for no impact on antimicrobial management** |
|---|---|---|---|
| **CAP (BAL fluid); CAP** | *Klebsiella pneumoniae; Pseudomonas aeruginosa* | *Achromobacter xylosoxidans* | long turnaround time |
| **Osteomyelitis (bone); Osteomyelitis** | *Escherichia coli; Pseudomonas aeruginosa* | *Bacteroides fragilis* | non-infection/ colonization |
| **HAP/VAP (BAL fluid); ILD progression** | *Klebsiella pneumoniae; Enterobacter cloacae* complex | *Bacteroides fragilis* | non-infection/ colonization |
| **Lung abscess (BAL fluid); Lung abscess** | *Klebsiella pneumonia* | *Mycoplasma* spp. | non-infection/ colonization |
| **SSI (tissue), SSI** | *Citrobacter freundii; Streptococcus mitis* | *Prevotella oris* | long turnaround time |

Abbreviations: BAL, bronchoalveolar lavage; CAP, community-acquired pneumonia; ILD, interstitial lung disease; HAP/VAP, Hospital-acquired pneumonia/ventilator-associated pneumonia; SSI, skin and soft tissue infection.
